# Evaluation of Antibiotic Resistance of *Salmonella* Serotypes and Whole-Genome Sequencing of Multiresistant Strains Isolated from Food Products in Russia

**DOI:** 10.3390/antibiotics11010001

**Published:** 2021-12-21

**Authors:** Andrey L. Rakitin, Yulia K. Yushina, Elena V. Zaiko, Dagmara S. Bataeva, Oksana A. Kuznetsova, Anastasia A. Semenova, Svetlana A. Ermolaeva, Aleksey V. Beletskiy, Tat’yana V. Kolganova, Andrey V. Mardanov, Sergei O. Shapovalov, Timofey E. Tkachik

**Affiliations:** 1 Research Center of Biotechnology, Institute of Bioengineering, Russian Academy of Sciences, 119071 Moscow, Russia; rakitin@biengi.ac.ru (A.L.R.); mortu@yandex.ru (A.V.B.); moldiag@biengi.ac.ru (T.V.K.); mardanov@biengi.ac.ru (A.V.M.); 2 V.M. Gorbatov Federal Research Center for Food Systems of Russian Academy of Sciences, 109316 Moscow, Russia; e.zaiko@fncps.ru (E.V.Z.); d.bataeva@fncps.ru (D.S.B.); o.kuznecova@fncps.ru (O.A.K.); a.semenova@fncps.ru (A.A.S.); 3 Federal Research Center for Virology and Microbiology, Nizhny Novgorod Research Veterinary Institute Branch, 603950 Nizhny Novgorod, Russia; drermolaeva@mail.ru; 4 Gamaleya National Research Centre for Epidemiology and Microbiology, 123098 Moscow, Russia; 5 Research and Scientific Testing Center “Cherkizovo”, 108805 Moscow, Russia; shapovalov73@rambler.ru (S.O.S.); tim.tkachik@gmail.com (T.E.T.)

**Keywords:** *Salmonella*, foodborne pathogen, serotype, WGS, antimicrobial resistance

## Abstract

Food products may be a source of *Salmonella*, one of the main causal agents of food poisoning, especially after the emergence of strains resistant to antimicrobial preparations. The present work dealt with investigation of the occurrence of resistance to antimicrobial preparations among *S. enterica* strains isolated from food. The isolates belonged to 11 serovars, among which Infantis (28%), Enteritidis (19%), and Typhimurium (13.4%) predominated. The isolates were most commonly resistant to trimethoprim/sulfamethoxazole (*n =* 19, 59.38%), cefazolin (*n =* 15, 46.86%), tetracycline (*n =* 13, 40.63%), and amikacin (*n =* 9, 28.13%). Most of the strains (68.75%) exhibited multiple resistance to commonly used antibiotics. High-throughput sequencing was used to analyse three multidrug-resistant strains (resistant to six or more antibiotics). Two of them (SZL 30 and SZL 31) belonged to *S.* Infantis, while one strain belonged to *S.* Typhimurium (SZL 38). Analysis of the genomes of the sequenced strains revealed the genes responsible for antibiotic resistance. In the genomes of strains SZL 30 and SZL 31 the genes of antibiotic resistance were shown to be localized mostly in integrons within plasmids, while most of the antibiotic resistance genes of strain SZL 38 were localized in a chromosomal island (17,949 nt). Genomes of the *Salmonella* strains SZL 30, SZL 31, and SZL 38 were shown to contain full-size pathogenicity islands: SPI-1, SPI-2, SPI-4, SPI-5, SPI-9, SPI-11, SPI-13, SPI-14, and CS54. Moreover, the genome of strain SZL 38 was also found to contain the full-size pathogenicity islands SPI-3, SPI-6, SPI-12, and SPI-16. The emergence of multidrug-resistant strains of various *Salmonella* serovars indicates that further research on the transmission pathways for these genetic determinants and monitoring of the distribution of these microorganisms are necessary.

## 1. Introduction

Food safety is a key problem worldwide, while the provision of pathogen-free food is an important social problem. Food pathogens are among the major food-related risk factors, affecting over 2 billion people per year [1]. *Salmonella* strains are among the major causes of foodborne diseases in many regions worldwide [2]. *Salmonella* is common among wild and domesticated animals, including the animals used by humans for food. While over 2600 *Salmonella* serotypes are presently known, almost 99% of the serotypes associated with diseases of humans and warm-blood animals belong to *S.* *enterica* subspecies *enterica* [3].

In the Russian Federation, salmonellosis remains an important cause for outbursts, being third in the nidus structure of group disease incidence among infections with a faecal-oral transmission mechanism. In 2020, 20 outbreaks of salmonellosis were recorded with a total of 422 people affected [4]. In cases of this disease, the strategy involves determination of the medically important serovars and of their sources in order to undertake relevant measures to limit their propagation [5].

In humans, *Salmonella*-infected foods usually cause an enteric disease which is cured by itself and, as a rule, does not require serious medical interference [6]. However, a severe form of this disease, occurring in 2–3% of all cases, may result in sepsis and other system disorders, especially in children, senior citizens, and people with impaired immunity. In these situations, antibiotic treatment may be an important life-saving measure [7,8]. Apart from their pathogenic potential, *Salmonella* strains may develop resistance to one or several antibiotics, which may hinder medical treatment. Unjustified application of antibiotics in livestock farming may favour the emergence of strains with multidrug resistance; this resistance may be transmitted by mobile genetic elements.

Reports on *Salmonella* resistance to antimicrobial preparations, including cases of multiple resistance, with three or more preparations inefficient against a certain strain, have become more frequent with time [9,10,11].

Full-genome sequencing is presently recommended for characterization of microorganisms, since it provides extensive information, including serovar distribution, profiles of the virulence and antibiotic resistance genes, and the presence of plasmids. It also makes possible multilocus typing using the sequences obtained [12].

The NCBI GenBank database presently contains over 12,500 full-size sequences of the species *S.* *enterica*. Numerous works on *Salmonella* genomes deal with the detection of genetic loci related to pathogenesis: antibiotic resistance genes, which are often located in integrons or transposons; *Salmonella* pathogenicity islands (SPI); and bacteriophages [13,14,15,16].

Microbial resistance to antibiotics may be caused by several mechanisms [17,18,19]: (1) inactivation of an antibiotic by its enzymatic modification via phosphorylation, adenylation, or acetylation; (2) modification of the antibiotic target site; (3) release of antibiotic from the cell by various transporters; and (4) decreased membrane permeability.

The aim of this work was to study the antimicrobial resistance of various serotypes of *Salmonella* derived from food. Genome-wide sequencing of multiresistant strains was carried out in order to study the determinants of antibiotic resistance, pathogenicity islands, and phages localized in the genomes of these strains.

## 2. Results

### 2.1. Prevalence of Salmonella spp. in Food Products

Of the 474 samples, 32 (6.8%) were positive for *Salmonella* spp.: 12 poultry samples (10.7%), 3 pork samples (3.3%), 7 beef samples (6.8%), and 10 minced meat samples (6%) (Table 1).

### 2.2. Serological Identification of Salmonella

**Figure 1:**
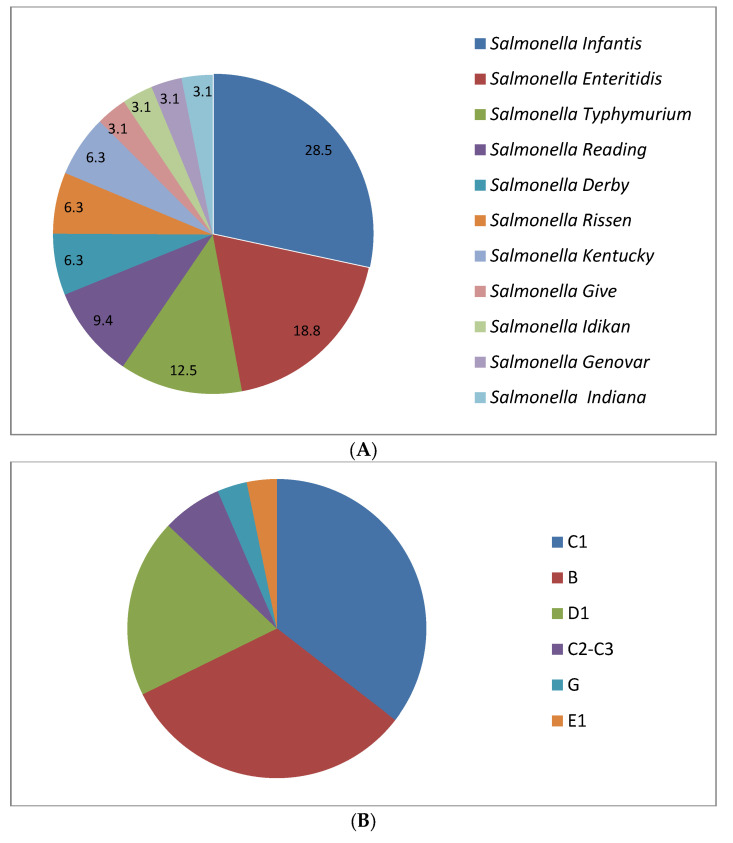
(**A**) Frequency of occurrence of *Salmonella* serotypes (*n =* 32). All isolates belonged to 11 serovars. The share (%) of each serovar is represented as a ring diagram. (**B**) Categories of serogroups based on the serovars. Serogroup C_1_: Rissen and Infantis. Serogroup B: Derby, Typhimurium, Indiana, and Reading. Serogroup D_1_: Enteritidis. Serogroup C_2_–C_3_: Kentucky. Serogroup G: Idikan. Serogroup E_1_: Give.

The studied isolates belonged to 11 serovars. The most common serovars were Infantis (28.13%, *n =* 9), then Enteritidis (18.75%, *n =* 6), Typhimurium (12.50%, *n =* 4), Reading (9.38%, *n =* 3), Derby, Rissen, and Kentucky (6.25%, *n =* 2), and Give, Idikan, Genovar, and Indiana (3.13%, *n =* 1) (Figure 1).

The serovars were distributed among the different types of sample, indicating high genetic diversity of *Salmonella* strains occurring in the Russian Federation.

In general, these serovars fell into six serogroups (C_1_, B, D_1_, C_2_–C_3_, G, and E_1_). Serogroup C_1_ (35.48%, *n =* 11) was the most common, followed by B (32.26%, *n =* 10), D_1_ (19.35%, *n =* 6), C_2_–C_3_ (6.45%, *n =* 2), G (3.23, *n =* 1), and E_1_ (3.23%, *n =* 1).

### 2.3. Prevalence of Antimicrobial Resistance

Research on the sensitivity of *Salmonella* isolates to antibiotics revealed various levels of resistance to 13 antimicrobial preparations (Table 2). The isolates were most commonly resistant to trimethoprim/sulfamethoxazole (*n =* 19, 59.38%), cefazolin (*n =* 15, 46.86%), tetracycline (*n =* 13, 40.63%), and amikacin (*n =* 9, 28.13%). They were most sensitive to amoxicillin–clavulanic acid (*n =* 30, 93.75%), tobramycin (*n =* 26, 81.25), ampicillin (*n =* 23, 71.88%), and azithromycin (*n =* 20, 62.50%).

Resistance to antimicrobial preparations varied between serotypes (Table 3). In general, resistance was higher among the *Salmonella* serotypes often causing salmonellosis in humans, rather than in less common serotypes. *S.* Typhimurium was most often resistant to streptomycin (75%), trimethoprim/sulfamethoxazole (75%), tetracycline (75%), azithromycin (50%), and cefazolin (50%); all strains were, however, resistant to amikacin and amoxicillin + clavulanic acid. *S.* Enteritidis isolates were resistant to cefazolin (50%), trimethoprim/sulfamethoxazole (50%), azithromycin (33%), and tetracycline (33%); at the same time, all strains were sensitive to streptomycin, tobramycin, amoxicillin + clavulanic acid, and nitrofurantoin. The most represented serotype, Infantis, was resistant to all antibiotics tested. In *S.* Infantis isolates, the highest percentage of resistance was shown for trimethoprim/sulfamethoxazole (66.7%) and tetracycline (55.6%). Resistance to cephalosporins was also revealed. The highest resistance to cefazolin was observed among *S.* Give (100%), *S.* Reading (66.7%), *S.* Typhimurium (50%), *S.* Enteritidis (50%), and *S.* Kentucky (50%). *S.* Derby (50.0%), *S.* Enteritidis (33.3%), and *S.* Reading (33.3%) were most often resistant to cefotaxime.

In total, 30 resistance patterns of these *Salmonella* isolates to eight groups of antimicrobial agents were found (Table 4).

Table 3 shows that only two of the studied strains (6.25%) were sensitive to all tested antimicrobial preparations. The remaining 93.75% were resistant to at least one class of antimicrobial preparation, while 68.75% of the strains exhibited multiple resistance to three or more classes of antimicrobial agents. Among the studied isolates, 15.63% (5/32) were resistant against four groups of antimicrobial agents, while 25% (8/32) exhibited resistance to four individual preparations.

Three of the studied isolates exhibited the highest resistance. SZL 38 was resistant to six antibiotics belonging to four groups of antimicrobial compounds, while SZL 30 and SZL 31 were resistant to eight of the antimicrobial compounds belonging to six groups.

### 2.4. Sequencing

Sequencing was carried out for three multiply resistant strains: *S. enterica* serovar Infantis isolates SZL 30 and SZL 31, and *S.* Typhimurium SZL 38, which exhibited resistance to at least six antibiotics. The technologies used were Illumina (Illumina, San Diego, CA, USA) and single-molecular nanopore sequencing (Oxford Nanopore, Oxford, UK). The results for sequencing of genomic DNA libraries of the studied *Salmonella* strains are presented in Supplementary Table S1.

Assembling the contigs for each strain resulted in a complete circular chromosome sequence and a set of ring-shaped contigs representing the plasmids. Thus, the chromosome size of the sequenced strains was from 4.68 to 5.05 × 10^6^ nt, while the number of plasmids varied from one to two, depending on the strain (Table 5).

### 2.5. General Characterization of the Genomes

Analysis of the genomes of *Salmonella* strains SZL 30, SZL 31, and SZL 38 revealed that they contained 5078 to 5209 protein-encoding genes, with 73% of them being genes with predicted function. All sequenced genomes contained eight copies of 5S rRNA, seven copies each of 16S rRNA and 23S rRNA, and 84 to 89 tRNA (Table 6).

### 2.6. Multilocus Sequence Typing of Strains

From analysis of the genomes of the *Salmonella* strains SZL 30, SZL 31, and SZL 38 using the Salmonella In Silico Typing Resource (SISTR) and SeqSero2, it was established that SZL 30 and SZL 31 belong to the serovar Infantis, and SZL 38 to the serovar Typhimurium.

### 2.7. Antibiotic Resistance Genes

Genome analysis of *Salmonella* strains SZL 30, SZL 31, and SZL 38 using the CARD and ResFinder 4.1 databases revealed that these strains possessed genes of resistance to streptomycin (*aadA1*—SZL 30, *aadA2b*—SZL 31, *aph(3″)-Ib* and *aph(6)-Id*—SZL 38), ampicillin, amoxicillin, cefazolin, piperacillin, and ticarcillin (*bla_TEM-1B_*), amikacin and tobramycin (*aac(6′)-Iaa*), sulfamethoxazole (*sul3*—SZL 30 and SZL 31; *sul2*—SZL 38), trimethoprim (*dfrA14*—SZL 30 and SZL 31), doxycycline and tetracycline (*tet(A)*—SZL 30 and SZL 31; *tet(B)*—SZL 38), chloramphenicol (*cmlA1*), rifampicin, erythromycin, and azithromycin (*mefB*—SZL 30 and SZL 31), puromycin and erythromycin (*mdfA*/*cmr*), novobiocin, nalidixic acid, and norfloxacin (*mdtABC*-*tolC*).

### 2.8. Plasmids

*Salmonella* strain SZL 30 possessed an F-type conjugative plasmid pSZL30.1 (276,251 nt), bearing the genes of antibiotic resistance *dfrA14* and *tetA*. pSZL30.1 belongs to the IncFIB incompatibility group and contains four toxin–antitoxin loci (*ccdA*/*ccdB*, *vapB*/*vapC*, *relB*/*relE*, and *pemI*/*pemK*), which are responsible for its stable inheritance in the population due to the mechanism of post-segregational killing. The conjugative Ti-type plasmid pSZL30.2 (53,986 nt) contains the *sat1*, *cmlA1*, *aadA1*, *sul3*, and *bla_TEM-1B_* resistance genes, belongs to the IncX1 incompatibility group, and contains the *relB*/*relE* toxin–antitoxin locus.

The *Salmonella* strain SZL 31 possessed an F-type conjugative plasmid pSZL31.1 (280,239 nt), bearing the *sat1*, *cmlA1*, *sul3*, *tetA*, *aadA2b*, and *dfrA14* genes of antibiotic resistance. pSZL31.1 belongs to the IncFIB incompatibility group and contains four toxin–antitoxin loci (*ccdA*/*ccdB*, *vapB*/*vapC*, *relB*/*relE*, and *pemI*/*pemK*). The Ti-type conjugative plasmid pSZL31.2 (53,147 nt) of the IncX1 incompatibility group contains the *relB*/*relE* toxin–antitoxin locus and bears the genes of antibiotic resistance *cmlA1*, *sul3*, and *bla_TEM-1B_*.

Strain *Salmonella* SZL 38 possessed the plasmid pSZL38.1 belonging to the p0111 incompatibility group and containing the *relB*/*relE* toxin–antitoxin locus. Comparison of the nucleotide sequence of pSZL38.1 to GenBank sequences revealed 98.84% homology to pD72C of *E. coli* strain D72C [20], which may indicate horizontal gene transfer in the family *Enterobacteriaceae*.

### 2.9. Pathogenicity Islands

Comparison of the nucleotide sequences of pathogenicity islands [21,22,23] with the genomes of the sequenced strains revealed that the genomes of *Salmonella* strains SZL 30, SZL 31, and SZL 38 contained the following full-sized pathogenicity islands: SPI-1, SPI-2, SPI-4, SPI-5, SPI-9, SPI-11, SPI-13, SPI-14, and CS54 (Supplementary Table S2). The genome of strain SZL 38 also contained the full-sized pathogenicity islands SPI-3, SPI-6, SPI-12, and SPI-16, while the genomes of strains SZL 30 and SZL 31 lacked some of the genes involved in the above pathogenicity islands (Supplementary Table S3). Pathogenicity islands SPI-7, SPI-8, SPI-10, SPI-15, SPI-18, SPI-19, SPI-20, SPI-21, SPI-22, and SPI-23 were not found in the genomes of *Salmonella* strains SZL 30, SZL 31, and SZL 38.

### 2.10. Prophages

Analysis of the presence of prophages in the sequenced genomes using the PHASTER software package revealed 14 prophages in the genome of *Salmonella* SZL 30 and 13 prophages in the genomes of both *Salmonella* SZL 31 and *Salmonella* SZL 38 (Supplementary Table S2). The prophages SZL 30-1, SZL 30-2, SZL 30-3, SZL 30-4, SZL 31-1, SZL 31-2, SZL 31-3, SZL 31-4, SZL 38-1, SZL 38-1, SZL 38-2, SZL 38-3, SZL 38-4, SZL 38-5, and SZL 38-6 were intact, while the remaining ones were dubious or incomplete (Supplementary Table S3).

Comparison of the sequences of these prophages with the GenBank data revealed most of them to be widespread in the genomes of previously sequenced *Salmonella* strains. The sequences of 32 out of 40 revealed prophages exhibiting over 99% homology to the database sequences at coverage of over 99% (Supplementary Table S3).

## 3. Discussion

Salmonellosis is considered a serious public healthcare issue worldwide [24]. The emergence of *Salmonella* strains with multiple drug resistance is an important problem in terms of human health and may result in inefficient treatment [25]. *Salmonella* spp. are among the main pathogens causing food poisoning in the Russian Federation and the third most important ones in the structure of the nidi of group diseases with faecal-oral transmission mechanisms. In 2020, 20 outbreaks of salmonellosis group infection were revealed (compared to 70 in 2019), which affected 422 people [4].

The overall prevalence of *Salmonella* in this study was 6.8%. In general, the high isolation rate of *Salmonella* spp. in chicken samples indicates that chicken is one of the most important sources of human salmonellosis.

In the present work, 32 bacterial isolates were obtained and identified as *Salmonella* strains based on their biochemical characteristics. The most common serovar was Infantis (28.13%, *n =* 9). *S.* Infantis is considered one of the most frequent causes for bacterial food poisoning worldwide [26,27]. Its high occurrence (25.8%) was revealed by serotyping of the *Salmonella* isolates from food carried out in Turkey [28].

The second most common serovar was Enteritidis (18.75%, *n =* 6). *S.* *enterica* serotype Enteritidis (*S.* Enteritidis) is the most common *Salmonella* serotype worldwide, especially in retail meat products [29,30] and seafood [31]. Recent reports from Brazil, Poland, Malaysia, China, and Greece showed that Enteritidis was the dominant serovar with frequency of occurrence from 34% to 86%, which indicates a broad distribution worldwide [32,33,34,35].

The third most common serovar was Typhimurium. Among the major serovars, *S.* Typhimurium is mostly responsible for diarrhoea in humans [36]. In the present work, four isolates were identified as *S.* Typhimurium. This serotype was recently acknowledged as a new healthcare problem, since it has been isolated from various animals, environmental objects, and food; moreover, the frequency of human diseases caused by this organism in various countries is increasing [37,38,39].

These results are in complete agreement with the data from the report by the Russian Federal Service for Surveillance on Consumer Rights Protection and Human Wellbeing, since in 2015–2020 the predominant serotypes in Russian territory were *S.* Enteritidis (64.7%), *S.* Typhimurium (4.8%), and *S.* Infantis (3.2%). *S.* Infantis was most common in chicken meat, *S.* Kentucky in turkey meat, and *S.* Typhimurium in environmental objects and pork [4].

In most cases, salmonellosis is cured by itself, with antimicrobial therapy required only for invasive or long-term infections. However, large-scale application of antibiotics results in the development of resistance to antimicrobial preparations and favours the emergence of strains possessing multiple drug resistance (MDR) [40,41,42].

The *Salmonella* strains isolated in our work were tested for their sensitivity to 13 antimicrobial preparations significant for healthcare and veterinary medicine. Only two of the studied strains (6.25%) were sensitive to all tested antimicrobial agents. In total, 93.75% of the isolates were resistant to at least one class of antimicrobial preparations, while 68.75% exhibited MDR to three or more classes of antimicrobial agents. Multiple resistance implies simultaneous resistance to three or more antibiotics. Other researchers have also reported a high percentage of antibiotic-resistant *Salmonella* strains isolated from food [43,44,45]. Animals are the main reservoir of *Salmonella*, and irresponsible application of antimicrobial preparations to cure productive animals, for disease prevention, and for growth stimulation results in the emergence of resistant pathogens. Increased occurrence of human salmonellosis caused by food infected with *Salmonella* strains resistant to antimicrobial agents has been reported [46,47].

Investigation of the sensitivity of our *Salmonella* isolates to antimicrobial preparations showed that the greatest number of isolates were resistant to folate pathway antagonists (trimethoprim/sulfamethoxazole), followed by cephems (cefazolin) and the tetracycline group (tetracycline). High resistance to these groups of antibiotics is in agreement with previous reports [48,49], which emphasized uncontrolled application of these antibacterial preparations. Resistance of 31 *Salmonella* strains isolated from poultry samples in Ethiopia was 60% [48]. Most studied isolates were sensitive to amoxicillin/clavulanic acid, imipenem, ampicillin, and streptomycin.

All *S.* Infantis isolates were resistant to at least one antibiotic, with the highest resistance observed for trimethoprim/sulfamethoxazole, tetracycline, chloramphenicol, and cefazolin. The *S.* Enteritidis isolates were most often resistant to trimethoprim/sulfamethoxazole, cefazolin, azithromycin, and tetracycline.

*S.* Typhimurium was most resistant to streptomycin, trimethoprim/sulfamethoxazole, tetracycline, and azithromycin. All studied *S.* Typhimurium and *S.* Enteritidis strains were sensitive to nitrofurantoin and amoxicillin + clavulanic acid.

Our findings of high resistance to trimethoprim/sulfamethoxazole are similar to the results obtained previously [50] for *S. enterica* serovars isolated in Egypt from broiler chickens and chicken carcasses, as well as for *Salmonella* isolates from chicken carcasses sold in Ibagué, Columbia [51].

The high levels of antibiotic resistance of *Salmonella* food isolates revealed in the present work indicate non-selective and constant application of higher than therapeutic doses of antibiotics to animals.

Antimicrobial resistance (AMR) poses a colossal threat to global health and incurs high economic costs to society. This ever-growing problem not only threatens public health, but also incurs a huge toll on a nation’s economic growth by delayed hospitalizations, lengthening recovery time, expensive medicines, and specialized care for patients [10,11,12]. Authors have estimated the economic cost of resistance per antibiotic by drug class and compared those in developing and developed countries, like in Thailand and the United States [52]. The total economic cost of AMR due to resistance in five pathogens, *S. aureus*, *E. coli*, *K. pneumoniae*, *A. baumannii*, and *P. aeruginosa* was $0.5 billion and $2.9 billion in Thailand and the US, respectively. So, resistance has a significant impact on the cost of treatments [11]. Increased illness coupled with limited options for treatment further strains low-income settings already struggling with low resources.

Antibiotic-resistant infections also affect animals, and the increasing rates of resistance also mean that it becomes more difficult to treat such infections. Death to livestock can further damage the finances of both individual citizens and society. Novel treatments for MDR infections can cost up to tens of thousands of US dollars, which ultimately makes the medicines unreachable for many [6]. Understanding the depth of the problem of the prevalence of resistance, especially multiple resistance, primarily among pathogenic microorganisms can result in better-informed policy recommendations regarding interventions that affect antimicrobial consumption and those aimed specifically at reducing the burden of AMR.

In the second stage of the work, *Salmonella* strains selected in the previous stage (SZL 30, SZL 31, and SZL 38) were sequenced. Genomic analysis of the sequenced strains revealed the genes responsible for antibiotic activity, and their localization was determined.

The *Salmonella* strains SZL 30, SZL 31, and SZL 38 were found to be streptomycin-resistant (Table 3).

Aminoglycoside resistance in *Salmonella* is also related to the expression of drug-modifying enzymes. These enzymes are classified into three main groups, according to the type of reaction they catalyse: acetyltransferases, encoded by the *aac* genes, phosphotransferases encoded by *aph* genes, and nucleotidyltransferases, encoded by *aadA* genes. Many variations of genes in each class are described [53]. In this study, all the strains had at least one of the three classes of genes described.

Strain SZL 38 had two different genes associated with resistance to aminoglycosides localized in chromosomes. Previously, it has been reported that there are different variants of resistance to aminoglycosides in the same strains isolated from the poultry production chain [42,51]. This may be due to the intense selection pressure in these production lines.

Resistance of *Salmonella* strain SZL 30 was probably caused by the *aadA1* gene localized in the plasmid pSZL30.2 and encoding aminoglycoside-3′-adenylyl transferase, which inactivates streptomycin or spectinomycin by adenylation (Table 7). The nucleotide sequence of *aadA1* was 100% identical to the previously described *aadA1* gene (JQ414041.1) from *Pseudomonas aeruginosa* strain K7 [54]. Streptomycin resistance of *Salmonella* strain SZL 31 may be due to the presence of the *aadA2b* gene localized in the plasmid pSZL31.1 and also encoding aminoglycoside-3′-adenylyl transferase. The nucleotide sequence of the previously described *aadA2b* gene (D43625.1) from the plasmid pSA1700 of *P. aeruginosa* strain ST1700, which provides *Escherichia coli* cells with streptomycin resistance [55], differs by one pointwise replacement from the *aadA2b* gene of the plasmid pSZL31.1. Streptomycin resistance of *Salmonella* strain SZL 38 may be caused by the presence of the *aph(3″)-Ib* and *aph(6)-Id* genes, encoding aminoglycoside phosphotransferases, which inactivate a number of aminoglycoside antibiotics by phosphorylation, in the chromosome of this strain. The nucleotide sequence of the *aph(3″)-Ib* gene was 100% identical to that of the previously described *aph(3″)-Ib* gene (AF321551.1) from the plasmid pSTR1 of *Shigella* *flexneri* strain 2731 [56]. The nucleotide sequence of the *aph(6)-Id* gene was 100% identical to that of the previously described *aph(6)-Id* gene (M28829.1) from the plasmid pRSF1010 of an *E. coli* strain [57].

The formation of genetic resistance to streptomycin in all three strains may be associated with the intensive use of streptomycin in animal husbandry, as evidenced by the results of a study of 66 meat samples of slaughter animals, in which a relatively high concentration of streptomycin was detected—5 × 10^−1^ (from 3 × 10^−1^ to 8 × 10^−1^) mg/kg at a limit of 0.2 mg/kg in the territory of the Russian Federation.

Resistance of *Salmonella* strains SZL 30, SZL 31, and SZL 38 to β-lactam antibiotics may be caused by the presence in the genomes of these strains of the *bla_TEM-1B_* gene, which is localized in the plasmids pSZL30.2 and pSZL31.2 and in the chromosome of strain SZL 38 (Table 7). The *bla_TEM-1B_* gene encodes β-lactamase, which provides bacterial resistance to β-lactam antibiotics by hydrolysing them. The nucleotide sequences of all revealed *bla_TEM-1B_* genes were 100% homologous to each other and to the previously described *bla_TEM-1B_* gene (HM769901.1) from the plasmid pZM3 of an *S. enterica* subsp. *enterica* serovar Wien strain [58]. β-Lactamase hydrolyses the β-lactam ring, resulting in the formation of β-amino acids that do not have antimicrobial activity [59]. The presence of genetic resistance to lactamases, which are often used to treat humans, among *Salmonella* strains isolated from meat products indicates their use as a prophylaxis or for the treatment of animals [60]. Such strains circulating in animals can infect humans and transmit antibiotic resistance to other pathogens [61].

Resistance of *Salmonella* strains SZL 30, SZL 31, and SZL 38 to amikacin and tobramycin was probably caused by the presence of the *aac(6’)-Iaa* gene in the chromosomes of these strains. The *aac(6’)-Iaa* gene encodes aminoglycoside-6′-acetyl transferase, which provides bacterial resistance to a number of aminoglycoside antibiotics by their acetylation. The nucleotide sequences of the *aac(6’)-Iaa* genes located in the chromosomes of the *Salmonella* strains SZL 30 and SZL 31 were 100% identical and 97.5% homologous to the previously described *aac(6’)-Iaa* gene (NC_003197.2) from *Salmonella enterica* subsp. *enterica* serovar Typhimurium str. LT2 [62]. The sequence of the *aac(6’)-Iaa* gene localized in the chromosome of *Salmonella* strain SZL 38 was 100% identical to that of the previously described *aac(6’)-Iaa* gene (NC_003197.2), which is known to provide *E. coli* cells with resistance to amikacin, tobramycin, and kanamycin [63].

Sulphonamide resistance in Gram-negative bacilli usually results from the acquisition of one of three *sul1*, *sul2*, and *sul3* genes encoding forms of dihydropteroate synthase that are not inhibited by the drug. Resistance of the *Salmonella* strains SZL 30 and SZL 31 to sulfamethoxazole was due to the presence of the *sul3* gene localized in the plasmids pSZL30.1, pSZL31.1, and pSZL31.2 and encoding the sulfamethoxazole-resistant dihydropteroate synthase (Table 7). Nucleotide sequences of the *sul3* located in the plasmids pSZL30.1, pSZL31.1, and pSZL31.2 were 100% homologous to each other and to the previously described *sul3* gene (AJ459418) from the pVP440 plasmid of *E. coli* strain rl0044, providing *E. coli* cells with sulfamethoxazole resistance [64]. Sulfamethoxazole resistance of *Salmonella* strain SZL 38 may be due to the presence of the *sul2* gene encoding dihydropteroate synthase in the chromosome of this strain. The spread of the *sul2* gene has increased over the years in other European countries [2,9], as it has often been reported that this gene is more widespread among clinical isolates of *E. coli* than the *sul1* gene. The nucleotide sequence of the *sul2* gene was 100% homologous to that of the previously described *sul2* gene (HQ840942.1) for sulfamethoxazole resistance from the plasmid pSRC27-H of *S. enterica* strain SRC27 [65].

Trimethoprim resistance of *Salmonella* strains SZL 30 and SZL 31 was probably due to the presence of the *dfrA14* gene localized in the plasmids pSZL30.1 and pSZL31.1 and encoding the trimethoprim-resistant dihydrofolate reductase, which is responsible for synthesis of tetrahydrofolate from dihydrofolate (Table 7). The nucleotide sequences of the *dfrA14* located in the plasmids pSZL30.1 and pSZL31.1 were 100% homologous to each other and to the previously described *dfrA14* gene (AJ313522.1) from the plasmid pSTOJO1 of a uropathogenic *E. coli* strain, which provides trimethoprim resistance to *E. coli* cells [66].

Resistance of the *Salmonella* strains SZL 30 and SZL 31 to tetracycline may be caused by the presence of the *tet(A)* gene localized in the plasmids pSZL30.1 and pSZL31.1 and encoding the MSF transporter, providing resistance to the tetracycline group antibiotics by removing these compounds from the cell (Table 7). The *tet(A)* and *tet(B)* genes were more associated with tetracycline resistance, which is consistent with previous studies [6,14].

The nucleotide sequences of the *tet(A)* genes localized in the plasmids pSZL30.1 and pSZL31.1 were 100% homologous to each other and to the previously described *tet(A)* gene (AJ517790.2) from the plasmid pRAS1 of *Aeromonas salmonicida* strain 2402, which provides *E. coli* with resistance to the tetracycline group antibiotics [67,68]. Tetracycline resistance of *Salmonella* strain SZL 38 was probably caused by the presence in its chromosome of the *tet(B)* gene, which also encodes the MSF transporter, providing resistance to tetracycline group antibiotics by removing these compounds from the cell. The nucleotide sequence of the *tet(B)* gene differed by one pointwise replacement from the previously described *tetA(B)* gene (P02980.1) of tetracycline resistance from the Tn10 transposon [69].

*Salmonella* strains SZL 30 and SZL 31, unlike strain SZL 38, were found to be resistant to chloramphenicol (Table 7). Chloramphenicol resistance of the *Salmonella* strains SZL 30 and SZL 31 was probably caused by the presence of the *cmlA1* gene located in the plasmids pSZL30.1, pSZL31.1, and pSZL31.2 and encoding the MSF transporter of the Bcr/CflA family, which is responsible for bacterial resistance to chloramphenicol due to its release from the cell. The nucleotide sequences of the *cmlA1* genes localized in the plasmids pSZL30.1, pSZL31.1, and pSZL31.2 were 100% homologous to each other and to the previously described *cmlA1* gene (U12338.3) from the plasmid R1033 of a *P. aeruginosa* strain, which provides *E. coli* cells with chloramphenicol resistance [70].

Thus, as a result of our study, it was found that strains SZL 30 and SZL 31 are resistant to the following antibiotics: STR, AMP, AMK, TM, STX, IPM, TET, and CHL, which correlated with the identified genetic elements fixed in the genome or localized in plasmids. However, the revealed phenotypic resistance to imipenem has not been confirmed at the genetic level; it is possible that the genetic determinants of resistance to imipenems may be non-specific carrier genes. We have shown the phenotypic resistance of strain SZL 38 to the following antibiotics: streptomycin (STR), ampicillin (AMP), amikacin (AMK), tobramycin (TM), trimethoprim/sulfamethoxazole (STX), and tetracycline (TET). There was a 100% correlation between the phenotypic and genotypic data for strain SZL 38. From analysis of the genomes of these strains, genetic determinants were identified, presumably responsible for resistance to STR, AMP, AMK, and TET. The gene *dfrA14*, responsible for resistance to trimethoprim, was not found; it is possible that the genetic determinants of resistance to trimethoprim may be non-specific transporter genes.

The high prevalence of *aadA1*, *aadA2b*, *aph(3″)-Ib*, *bla_TEM-1B_*, *tet(A)*, *tet(B)*, and *cmlA1* genes in *Salmonella* isolates may be due to the common use of aminoglycosides, phenicols, β-lactams, sulphonamides, and tetracycline group antibiotics in animal husbandry to combat bacterial infections and stimulate growth [6]. The presence of these genes in mobile genetic elements, such as transposons and plasmids, can facilitate their transfer [33].

According to a study in the territory of the Russian Federation, the meat of productive animals contained aminoglycosides, phenicols, β-lactams, sulphonamides, and tetracycline group antibiotics in 36.7%, 23.2–30%, 13.3–16%, 23.2–29.6%, and 18.5% of the studied samples, respectively [71].

The *Salmonella* strains SZL 30, SZL 31, and SZL 38 were potentially resistant to puromycin and erythromycin (*mdfA*/*cmr*), as well as to novobiocin, nalidixic acid, and norfloxacin (*mdtABC*-*tolC*). *Salmonella* strains SZL 30 and SZL 31 were also resistant to rifampicin, erythromycin, and azithromycin (*mefB*) (Table 7). In the genomes of *Salmonella* strains SZL 30 and SZL 31, most antibiotic resistance genes were localized in plasmids.

**Figure 2:**
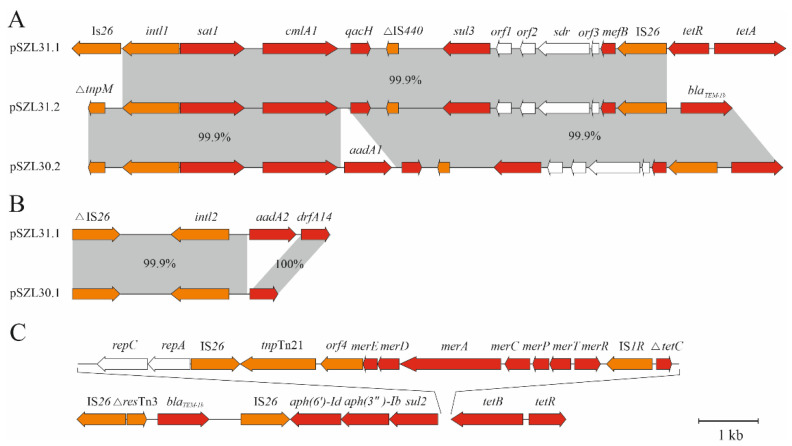
(**A**) Diagram of type I integrons and their flanking regions found in plasmids pSZL31.1, pSZL31.2, and pSZL30.2. (**B**) Schematic of type II integrons found in plasmids pSZL31.1 and pSZL30.1. (**C**) An island of resistance located on the chromosome of *Salmonella* S38. Red arrows indicate genes for resistance; orange genes are involved in the mobile transfer of genetic material. *aadA1*, *aadA2b*, *aph(3″)-Ib*, and *aph(6)-Id*—genes for streptomycin resistance, *bla_TEM-1B_*—ampicillin resistance gene, *cmlA1*—chloramphenicol resistance gene, *dfrA14*—trimethoprim resistance gene, *mefB*—gene for resistance to macrolide antibiotics, *qacH*—gene for resistance to quaternary ammonium compounds, *sul2* and *sul3*—genes for resistance to sulfamethoxazole, *sat1*—gene for resistance to streptothricin, *tetA* and *tetB*—genes for resistance to tetracycline, *tetR*—transcription regulator of genes *tetA* and *tetB*. merRTPCADE—mercury resistance operon. intl1 and intl2—integrases, IS*26* and IS*1R*—insertion elements, *orf1*, *orf2*, *orf3*, and *orf4*—genes coding proteins with unknown function, sdr—short chain dehydrogenase, *repA* and *repC*—replication proteins, *tnp*Tn*21*—transposase. ΔIS440, *ΔtetC*, *ΔtnpM*, and *Δres*Tn*3*—different variants of insertion element, transcription regulator, transposase, and resolvase, respectively.

The plasmid pSZL31.1 was 99.97% identical to pSZL30.1 at 96% coverage. The main differences between pSZL30.1 and pSZL31.1 were the presence or absence of various mobile genetic elements and antibiotic resistance genes. Thus, plasmid pSZL30.1, unlike pSZL31.1, contained the IS*2* and IS*903B* insertion elements. Unlike pSZL30.1, pSZL31.1 contained *tetA* with a type I integron, which had at the 3′ terminus the gene cassette (*sat1*, *cmlA*, and *qacH*) responsible for resistance to STR, chloramphenicol, and disinfectants (quaternary ammonium compound), which is coupled to the *sul3*–IS*26* compound element for sulphonamide resistance (Figure 2A). The plasmids pSZL30.1 and pSZL31.1 contained type II integrons. The integron of pSZL30.1 bears the *aadA2b* gene for spectinomycin/streptomycin resistance, while the integron of pSZL31.1, apart from *aadA2b*, also contains the *dfrA14* gene of trimethoprim resistance (Figure 2B).

At 98% coverage, the plasmid pSZL31.2 was 99.97% identical to pSZL30.2. pSZL30.2 and pSZL31.2 contained the gene *bla_TEM-1B_*, which had at the 3′ terminus a type I integron bearing the cassette of resistance genes coupled to the *sul3*–IS*26* element of sulphonamide resistance. While in pSZL30.2 the resistance cassette contained the genes *sat1*, *cmlA*, *aadA1*, and *qacH*, the cassette of pSZL31.2 contained the genes *sat1*, *cmlA*, and *qacH* (Figure 2A).

Comparison of the nucleotide sequences of the plasmids pSZL30.2 and pSZL31.2 with GenBank data revealed that they exhibited the highest homology (99.9%) at 66% coverage with an unnamed plasmid from *Shigella flexneri* strain 95-3008 (CP026773.1).

All antibiotic resistance genes detected in *Salmonella* strain SZL 38 were localized in the chromosome. Five genes, *bla_TEM-1B_*, *aph(6)-Id*, *aph(3″)-Ib*, *sul2*, and *tet(B)*, were localized in a 17,949 nt resistance island (Figure 2C). Comparison of the nucleotide sequences of this island with GenBank data revealed that it differed by one pointwise replacement from a similar island (CP019649.1) in the chromosome of *S. enterica* strain TW-Stm6 [72].

AMR is a serious and growing problem for *S. enterica* and other Gram-negative pathogens [73]. Overuse of antibiotics in medicine and agriculture is the most important factor contributing to the emergence of bacteria resistant to various antibiotics [74]. Poultry, cattle, and pigs can act as effective carriers of *Salmonella* to humans [75,76,77].

AMR can occur as a result of point mutations in the bacterial genome or as a result of the horizontal transfer of genetic elements carrying resistance genes. Probably the most effective way of transferring antibiotic resistance genes between microorganisms is the horizontal transfer of mobile genetic elements—integrons, transposons, and plasmids containing one or more genes that determine antibiotic resistance [78,79]. In our work, it was shown that strains SZL 30 and SZL 31 contain plasmids pSZL30.1, pSZL30.2, pSZL31.1, and pSZL31.2, carrying various genes for antibiotic resistance (Figure 2). Integrons that are part of these plasmids and carry genes for antibiotic resistance are widespread in various strains of *Salmonella*, *Proteus mirabilis*, and *E. coli*. It is likely that these antibiotic resistance genes can be transmitted to other Gram-negative microorganisms, especially as a result of selective pressure caused by the use of antibiotics in agriculture.

## 4. Materials and Methods

### 4.1. Strains Used in the Work

In total, 32 *Salmonella* strains were included in the study. This study concerns 32 *Salmonella* strains isolated from 443 samples (112 samples from different types of poultry, 91 samples of pork, 103 samples of beef, and 168 samples of minced meat) collected in 2019 (Supplementary Table S4).

### 4.2. Isolation and Confirmation of Salmonella Isolates

The preparation of samples, isolation, and identification of *Salmonella* was done according to techniques recommended by the International Organization for Standardization ISO 6579-1: 2017 [80]. Twenty-five grams from the skin of each sample was aseptically excised using a sterile scalpel then weighed and aseptically homogenized with 225 mL of sterile buffered peptone water (Oxoid, Basingstoke, UK) in a laboratory blender for 1 min and incubated at 37 °C for 24 h. One hundred microlitres from each pre-enrichment broth was inoculated into 10 mL of Rappaport–Vassiliadis broth (RV; Obolensk, Russian Federation) and incubated at 42 °C for 24 h. Then, 1 mL from each pre-enrichment broth was inoculated into 10 mL of Muller–Kauffmann tetrathionate–novobiocin broth (MKTTn; Oxoid, Basingstoke, UK) and incubated at 37 °C for 24 h. A loopful of each enriched broth was streaked onto two selective solid media: xylose–lysine–desoxycholate (XLD) agar (Oxoid, Basingstoke, UK) and bismuth sulphite agar (Oxoid, Basingstoke, UK) then the inoculated plates were incubated at 37 °C for 24 h. All presumptive colonies on XLD and bismuth sulphite agar were picked up and cultured onto nutrient agar plates (FBUN SSC PMB, Obolensk, Russian Federation) and incubated at 37 °C for 24 h to be subjected to further confirmation by biochemical and serological identifications. Specific colonies were confirmed by biochemical reactions using API 20E tests in accordance with the manufacturer’s instructions (BioMérieux, Craponne, France) and frozen at −86 °C in collections of the Gorbatov Federal Centre of Food Systems. When the work started, strains were revitalized and characterized bacteriologically and biochemically to support their identities again.

### 4.3. Serotyping of Salmonella Isolates

Serotyping of *Salmonella* isolates was carried out by the molecular genetic method, using Check & Trace Salmonella (Check-Points B.V., Wageningen, The Netherlands) according to the manufacturer’s method (details available at http://www.checkandtrace.com/, accessed on 21 July 2021).

### 4.4. Screening of the Isolates for Resistance to Antimicrobial Preparations

The sensitivity of *Salmonella* isolates to antimicrobial preparations was determined using the disk diffusion test on Muller–Hinton agar plates in accordance with the recommendations of the Clinical and Laboratory Standards Institute (CLSI) [81]. The following 12 antimicrobial preparations were tested: ampicillin (AMP) 10 µg, imipenem (IPM) 10 µg, amikacin (AMK) 10 µg, streptomycin (STR) 10 µg, tobramycin (TR) 10 µg, cefotaxime (CTX) 30 µg, cefazolin (CFZ) 30 µg, trimethoprim/sulfamethoxazole (SXT) 1.25/23.75 µg, chloramphenicol 30 µg, azithromycin 15 µg, amoxicillin–clavulanic acid (AMC) 20/10 µg, furadonin 300 µg, and tetracycline (TET) 30 µg. All antibiotic-containing paper disks were manufactured by the St. Petersburg Pasteur Institute, Russia. *E. coli* ATCC 25922 was used to control the quality of the research. The isolates were classified as sensitive, intermediate, or resistant according to CLSI [81]. The isolates resistant to three or more different classes of antimicrobial preparations were considered MDR.

### 4.5. DNA Isolation and Sequencing

LB medium (5 mL) was inoculated with the material from a single colony of *Salmonella* strain SZL 30, SZL 31, or SZL 38 and incubated for 16–20 h at 30 °C in a New Brunswick C-24 shaker incubator. The cells were separated on an Eppendorf MiniSpin centrifuge for 5 min at 10,000 rpm. Total DNA was isolated using a DNeasy PowerSoil Kit (Qiagen, Hilden, Germany) according to the manufacturer’s recommendations.

The *Salmonella* spp. genomes were sequenced using Illumina technology (Illumina, San Diego, CA, USA) and monomolecular nanopore sequencing (Oxford Nanopore, Oxford, UK).

Genomic DNA (200 ng) was homogenized in a Bioruptor UCD 200 sonicator (Diagenode, Denville, NJ, USA) for 10 min at the maximal power (5 cycles of 30 s on/90 s off). Paired DNA libraries (300 × 2) were obtained using a NEBNext^®^ Ultra™ II DNA Library Prep Kit (NEB) according to the manufacturer’s conditions. The number and quality of the libraries thus obtained were determined using a Bioanalyzer 2100 capillary electrophoresis system (Agilent, Santa Clara, CA, USA). The DNA libraries were sequenced on MiSeq (Illumina) using a MiSeq Reagent Kit v3 (600-cycle; Illumina).

Genomic DNA was also sequenced using a MinION device (Oxford Nanopore, Oxford, UK). DNA libraries were obtained using a Ligation Sequencing Kit 1 D (SQK-LSK109) system (Oxford Nanopore), omitting the initial stage of genomic DNA fragmentation and then following the manufacturer’s recommendations. Genomic libraries were sequenced on a MinION Flow Cell FLO-110.

### 4.6. Bioinformatic Techniques

The contigs from all Illumina and Nanopore reads were assembled using Unicycler v. 0.4.8 [82].

In the next stage, pairwise intersecting reads obtained using MiSeq (Illumina) were combined using flash [83], and low-quality read ends were removed with Sickle. Complete genome sequences were assembled from the reads obtained on MiSeq and MinION using Flye 2.7 [84] and were twice corrected by Illumina reads with Pilon 1.22 [85].

Identification of genes and theoretical prediction of their functions were carried out using the RAST server [86,87].

The search for genes homologous to genes of antibiotic resistance was carried out using Comprehensive Antibiotic Resistance Database (CARD) [88] and ResFinder 4.1 (80% identity and 60% gene coverage) [89]. The search for insertion sequences was carried out using the ISfinder server [90]. The plasmid type was determined using the Plasmid Finder 2.1 database [91]. The search for prophages was carried out using the Phage Search Tool Enhanced Release (PHASTER) server [92]. SPI presence and completeness were determined by comparison of the SPI nucleotide sequences [22,23] with the sequences of the genomes of *Salmonella* strains SZL 30, SZL 31, and SZL 38.

### 4.7. Nucleotide Sequence Accession Number

Whole genomes for *Salmonella enterica* were submitted in BioProject PRJNA774121, and BioSamples SAMN22551429, SAMN22551430, and SAMN22551506. The version described in this paper is the first version.

## 5. Conclusions

In conclusion, this study found a high incidence of MDR in various serotypes of *Salmonella* isolated from various types of food, including those commonly seen in infections in humans. Three strains with multiple AMRs (more than six) had different resistance genes with a heterogeneous distribution in the bacterial genome, which may indicate intense selection pressure during rearing and treatment of animals. Therefore, it is necessary to implement a plan to combat the abuse of antibiotics in veterinary medicine, as well as a system of epidemiological surveillance, including one based on whole-genome sequencing.

## Figures and Tables

**Table 1:** Prevalence of *Salmonella* isolated from food products.

Sample Type	Number of Samples Analysed	Number of Positive Samples (%)
Poultry	112	12 (10.7)
Pork	91	3 (3.3)
Beef	103	7 (6.8)
Minced meat	168	10 (6)
Total	474	32 (6.8)

**Table 2:** Antimicrobial resistance of *Salmonella* isolated from food products.

Antimicrobial Class	Antimicrobial Agent	No. of Strains (%)		
		Resistant (R)	Intermediate (I)	Susceptible (S)
Penicillins	Ampicillin (AMP)	8 (25.00)	8 (3.13)	23 (71.88)
Monobactams/carbapenems	Imipenem (IPM)	6 (18.75)	2 (6.25)	24 (75.00)
Aminoglycosides	Amikacin (AMK)	9 (28.13)	14 (43.75)	9 (28.13)
	Streptomycin (STR)	8 (25.00)	4 (12.50)	20 (62.50)
	Tobramycin	5 (15.63)	1 (3.13)	26 (81.25)
Cephems	Cefotaxime (CTX)	6 (18.75)	6 (18.75)	20 (62.50)
	Cefazolin (CFZ)	15 (46.86)	8 (25.00)	9 (28.13)
Folate pathway antagonists	Trimethoprim/ sulfamethoxazole (SXT)	19 (59.38)	0 (0)	13 (40.63)
	Chloramphenicol (CHL)	7 (21.88)	3 (9.38)	22 (68.75)
Macrolides and azalides	Azithromycin	12 (37.50)	-	20 (62.50)
β-Lactam/β-lactamase inhibitor combinations	Amoxicillin–clavulanic acid (AMC)	2 (6.25)	0 (0)	30 (93.75)
Nitrofuran	Furadonin	5 (18.75)	3 (9.38)	24 (75.00)
Tetracyclines	Tetracycline (TET)	13 (40.63)	9 (28.13)	10 (31.25)

Keys: ampicillin (AMP) 10 µg, imipenem (IPM) 10 µg, amikacin (AMK) 10 µg, streptomycin (STR) 10 µg, tobramycin 10 µg, cefotaxime (CTX) 30 µg, cefazolin (CFZ) 30 µg, trimethoprim/sulfamethoxazole (SXT) 1.25/23.75 µg, chloramphenicol 30 µg, azithromycin 15 µg, amoxicillin–clavulanic acid (AMC) 20/10 µg, furadonin 300 µg, and tetracycline (TET) 30 µg.

**Table 3:** Resistance of various *Salmonella* strains to antimicrobial preparations.

Antimicrobial Agent	No. of Resistance Isolates by Serotype (Resistance Rate, %)											
	*Salmonella* Typhimurium	*Salmonella* Enteritidis	*Salmonella* Infantis	*Salmonella* Kentucky	*Salmonella* Rissen	*Salmonella* Reading	*Salmonella* Give	*Salmonella* Derby	*Salmonella* Idikan	*Salmonella* Indiana	*Salmonella* Genovar	Total
Ampicillin (AMP)	25.0	16.7	33.3	0.0	50.0	0.0	100.0	0.0	0.0	100.0	0.0	25.0
Imipenem (IPM)	25.0	16.7	33.3	50.0	0.0	0.0	0.0	0.0	0.0	0.0	0.0	18.8
Amikacin (AMK)	0.0	16.7	33.3	0.0	50.0	66.7	0.0	50.0	0.0	100.0	0.0	28.1
Streptomycin (STR)	75.0	0.0	33.3	0.0	0.0	0.0	0.0	50.0	0.0	100.0	0.0	25
Tobramycin (TM)	25.0	0.0	22.2	0.0	0.0	0.0	0.0	50.0	0.0	100.0	0.0	15.6
Cefotaxime (CFX)	25.0	33.3	11.1	0.0	0.0	33.3	0.0	50.0	0.0	0.0	0.0	18.8
Cefazolin (CFZ)	50.0	50.0	44.4	50.0	50.0	66.7	100.0	50.0	0.0	0.0	0.0	50
Trimethoprim/sulfamethoxazole (STX)	75.0	50.0	66.7	50.0	50.0	33.3	100.0	50.0	0.0	100.0	100.0	59.34
Chloramphenicol (CHL)	25.0	16.7	44.4	0.0	50.0	0.0	0.0	0.0	0.0	0.0	0.0	21.9
Azithromycin (AZM)	50.0	33.3	22.2	0.0	50.0	33.3	0.0	50.0	100.0	0.0	100.0	34.4
Amoxicillin + clavulanic acid (AMK)	0.0	0.0	22.2	0.0	0.0	0.0	0.0	0.0	0.0	0.0	0.0	6.3
Nitrofurantoin (NIT)	0.0	0.0	44.4	0.0	0.0	33.3	0.0	0.0	0.0	0.0	0.0	15.6
Tetracycline (TET)	75.0	33.3	55.6	0.0	0.0	33.3	0.0	50.0	0.0	100.0	0.0	40.6

**Table 4:** Phenotypic antibiotic resistance profiles of *Salmonella* serotypes.

Serovar	Isolate	Pattern	No. of Antimicrobial Agents	No. of Classes
*Salmonella* Give	S1	AMP-STX-CFZ	3	3
*Salmonella* Typhimurium	S3	STR-AMP-TM-IPM-CHL	5	4
	S5	CTX-STX-AZM-TET-CFZ	5	4
	S14	STR-STX-AZM-TET-CFZ	5	4
	S25	STR-STX-TET	3	3
*Salmonella* Reading	S23	CTX-AMK-AZM-CFZ	4	3
	S4	AMK-STX-AZM-TET-NIT	5	5
	S33	CFZ	1	1
*Salmonella* Derby	S6	STR-AMK-AZM-TET	4	3
	S11	CTX-TM-STX-CFZ	4	3
*Salmonella* Idikan	S9	AZM	1	1
*Salmonella* Rissen	S10	CFZ-CHL	1	1
	S18	AMP-AMK-STX-AZM	4	4
*Salmonella* Infantis	S26	STR-IPM-CFZ-NIT	4	4
	S15	AMK-STX-AZM-TET-CFZ	5	5
	SZL 30	STR-AMP-AMK-TM-STX-IPM-TET-CHL	8	6
	SZL 31	STR-AMP-AMK-TM-STX-IPM-TET-CHL	8	6
	S34	AMK-STX-TET-CFZ-NIT	5	5
	S35	STX	1	1
	S36	ND	0	0
	S37	STX-TET-NIT	3	3
	S28	CTX-AZM-CFZ-CHL-NIT	5	4
*Salmonella* Genovar	S16	STX-AZM	2	2
*Salmonella* Enteritidis	S7	AMK-AZM-CFZ	3	3
	S8	CTX-AZM	2	2
	S17	AMP-STX-TET-CFZ-CHL	5	5
	S20	STX-IPM-CFZ	3	3
	S27	CTX-TET	2	2
	S29	STX	1	1
*Salmonella* Kentucky	S32	ND	0	0
	S19	STX-IMP-CFZ	3	3
*Salmonella* Typhimurium	SZL 38	STR-AMP-AMK-TM-STX-TET	6	4

Keys: ampicillin (AMP) 10 µg, imipenem (IPM) 10 µg, amikacin (AMK) 10 µg, streptomycin (STR) 10 µg, tobramycin 10 µg, cefotaxime (CTX) 30 µg, cefazolin (CFZ) 30 µg, trimethoprim/sulfamethoxazole (SXT) 1.25/23.75 µg, chloramphenicol 30 µg, azithromycin 15 µg, amoxicillin–clavulanic acid (AMC) 20/10 µg, furadonin 300 µg, and tetracycline (TET) 30 µg.

**Table 5:** Genomes of the *Salmonella* strains.

Strain	Chromosome Size (bp)	Plasmids	
		Name	Size (bp)
SZL 30	4,689,375	pSZL30.1 pSZL30.2	276,251 53,986
SZL 31	4,689,704	pSZL31.1 pSZL31.2	280,239 53,147
SZL 38	5,052,615	pSZL38.1	79,333

**Table 6:** General characteristics of the *Salmonella* genomes.

Parameter	Strain		
	30	31	38
Predicted genes	5184	5191	5320
Protein-coding genes	5078	5085	5209
Protein-coding genes with predicted function	3845 (75.7%)	3785 (74.4%)	3851 (73.9%)
tRNA genes	84	84	89

**Table 7:** Antibiotic resistance genes in the *Salmonella* genomes.

Resistance Gene	Protein	Antimicrobial Agent	Location		
			SZL 30	SZL 31	SZL 38
*aadA1*	Aminoglycoside (3″) (9)-adenylyltransferase	STR	pSZL30.2	-	-
*aadA2b*			-	pSZL31.1	-
*aph(3″)-Ib*	Aminoglycoside 3′-phosphotransferase		-	-	chromosome
*aph(6)-Id*	Aminoglycoside O-phosphotransferase		-	-	chromosome
*bla_TEM-1B_*	Class A β-lactamase	AMP	pSZL30.2	pSZL31.2	chromosome
*aac(6′)-Iaa*	Chromosomal encoded aminoglycoside N (6′)-acetyltransferase	AMK, TM	chromosome	chromosome	chromosome
*sul3*	Dihydropteroate synthase	STX	pSZL30.2	pSZL31.1 pSZL31.2	-
*sul2*			-	-	chromosome
*tet(A)*	Tetracycline efflux MSF transporter	TET	pSZL30.1	pSZL31.1	-
*tet(B)*			-	-	chromosome
*cmlA1*	Drug efflux MSF transporter Bcr/CflA family	CHL	pSZL30.2	pSZL31.1 pSZL31.2	-
*mefB*	MSF efflux transporter	AZM, ERY	pSZL30.2	chromosome pSZL31.2	-
*mdfA/cmr*	MSF efflux transporter	RIF, PUR, ERY	chromosome	chromosome	chromosome
*mdtABC*-*tolC*	MSF efflux transporter	NB, NAL, NOR	chromosome	chromosome	chromosome

## Data Availability

Data is contained within the article and Supplementary Materials.

## Supplementary Materials

The following are available online at https://www.mdpi.com/article/10.3390/antibiotics11010001/s1, Table S1: Sequencing statistics, Table S2: Pathogenicity Islands identified in the genomes of *Salmonella* strains, Table S3: Prophages identified in the genomes of *Salmonella* strains, Table S4: Bacterial strains used in the study.
