# Supplementary material for: Evaluation of Antibiotic Resistance of Salmonella Serotypes and Whole-Genome Sequencing of Multiresistant Strains Isolated from Food Products in Russia
Source: Antibiotics (Basel). 2021 Dec 21;11(1):1. doi: 10.3390/antibiotics11010001 (PMC8773070; doi:10.3390/antibiotics11010001)
Supplement: Supplementary file 1 [file antibiotics-11-00001-s001.zip › Supplementary Tables S1-S3.pdf]

Supplementary Table S1. Sequencing statistics

Strain	Illumina MiSeq		MinION	
	Reads	Average read length (nt)	Reads	Average read length (nt)
SZL 30	2482884	247	165065	6748
SZL 31	2324028	259	68614	6897
SZL 38	3294864	251	126596	7217

Supplementary Table S2. Pathogenicity Islands identified in the genomes of *Salmonella* strains.

SPI	Reference Strain			SZL 30		SZL 31		SZL 38	
	Serotype	Positon	Length (bp)	Positon	Length (bp) (Cowerage/ Identity)	Positon	Length (bp) (Cowerage/ Identity)	Positon	Length (bp) (Cowerage/ Identity)
SPI-1	Typhimurium LT2	3005849–3048153	42305	2217896-2260201	42306 (100/99)	1255831-1298136	42306 (100/99)	4145862-4188166	42305 (100/100)
SPI-2	Typhimurium LT2	1461731–1501480	39750	746128-785876	39749 (100/99)	2730163-2769911	39749 (100/99)	889254-929780	40527 (100/100)
SPI-3	Typhimurium LT2	3948999–3985278	36280	3185282-3216330	31049 (89/99) Missing genes: sugR, rhuM	300371-331419	31049 (89/99) Missing genes: sugR, rhuM	3334755-3370950	36196 (100/100)
SPI-4	Typhimurium LT2	4477865–4501275	23411	3727088-3750496	23409 (100/98)	4454845-4479318	24438 (100/98)	2819065-2842467	23403 (100/100)
SPI-5	Typhimurium LT2	1175536–1182100	6565	464191-470845	6655 (100/99)	3045194-3051848	6655 (100/99)	1209140-1215792	6652 (100/100)
SPI-6	Typhimurium	304666–	46693	4375990-	57525	3771823-	57525	2030954-	46662

	LT2	351358		4433514	(80/98) Missing genes: SirA, STM0275, STM0287, STM0289-STM0294, safA,	3829347	(80/98) Missing genes: SirA, STM0275, STM0287, STM0289-STM0294, safA,	2077615	100/100
SPI-7	Typhi CT18	4409652–4542913	133262	-	-	-	-	-	-
SPI-8	Typhi CT18	3132530–3139414	6885	-	-	-	-	-	-
SPI-9	Typhi CT18	2743495–2759190	15696	2097987-2114283	16297 (100/98)	1401753-1418049	16297 (100/98)	4520533-4536829	16297 (100/99)
SPI-10	Typhi CT18	4683605–4716538	32934	-	-	-	-	-	-
SPI-11	Typhimurium LT2	1326065–1334385	8321	613022-621331	8310 (100/99)	2894708-2903017	8310 (100/99)	1057126-1065446	8321 (100/100)
SPI-12	Typhimurium LT2	2330960–2345977	15018	1611395-1616573	5179 (34/99) Missing genes: ssrB, oafA, STM2233 - STM2240	1899462-1904640	5179 (34/99) Missing genes: ssrB, oafA, STM2233 - STM2240	4658-19668	15011 (100/100)
SPI-13	Typhimurium LT2	3276387–3301691	25305	2516654 - 2541953	25300 (100/99)	974751-1000050	25300 (100/99)	4415550-4440854	25305 (100/100)
SPI-14	Typhimurium LT2	926180–933609	7430	303154 - 310583	7430 (100/100)	3205457-3212886	7430 (100/100)	1407105-1414534	7430 (100/100)
SPI-15	Typhi CT18	3054094–3059809	5716	-	-	-	-	-	-
SPI-16	Typhimurium	613596–	4130	14918-	1265 (31/100)	3499866-	1265 (31/100)	1724278-1728407	4130 (100/100)

	LT2	617725		16182	Missing gene: STM0557	3501130	Missing gene: STM0557		
SPI-17	Typhi CT18	2461018–2465128	4111	-	-	-	-	-	-
SPI-18	Typhi CT18	1455055–1456801	1747	-	-	-	-	-	-
SPI-19	Dublin CT_02021853	1203281–1239108	35828	-	-	-	-	-	-
SPI-20	Arizonae 62:z4,z23	2617493–2651483	33991	-	-	-	-	-	-
SPI-21	Arizonae 62:z4,z23	2504768–2560524	55757	-	-	-	-	-	-
SPI-22	Bongori NCTC 12419	1349885-1369393	19,509	-	-	-	-	-	-
SPI-23	Derby 07CR553 LAZB01000005.1	1197720-1161181	36540	-	-	-	-	-	-
CS54	Typhimurium AF140550.2	1-25252	25252	1946303-1971555	25253 (100/97)	1544478-1569730	25253 (100/97)	4750278-4775529	25252 (100/100)

Supplementary Table S3. Prophages identified in the genomes of *Salmonella* strains.

Strain	Prophage	Completeness	Location	Position	Length, (kb)	The most similar Sequence NCBI GenBank (Coverage/Identity, %)
SZL 30	S30-1	intact	chromosome	1775254-1823074	47.8	CP052817.1 (84/98)
	S30-2	intact	chromosome	1244270-1276312	32	CP052817.1 (100/100)
	S30-3	intact	chromosome	3505067-3527042	21.9	CP052817.1 (100/100)
	S30-4	intact	pSZL30.1	23035-35898	12.8	CP052818.1 (100/100)
	S30-5	questionable	chromosome	2114772-2124257	9.4	CP052817.1 (100/100)
	S30-6	incomplete	chromosome	3666982-3687422	20.4	CP052817.1 (100/100)
	S30-13	incomplete	pSZL30.2	16350-32841	16.4	CP027138.1 (77/100)
	S30-7	incomplete	chromosome	1368812-1383062	14.2	CP052817.1 (100/100)

	S30-11	incomplete	pSZL30.1	100316-113967	13.6	CP040066.1 (93/100)
	S30-8	incomplete	chromosome	7926-18526	10.6	CP052817.1 (100/100)
	S30-9	incomplete	chromosome	1754042-1762443	8.4	CP052817.1 (100/100)
	S30-10	incomplete	chromosome	2259677-2267561	7.8	CP052817.1 (100/100)
	S30-14	incomplete	pSZL30.2	7049-14803	7.7	CP027138.1 (87/100)
	S30-12	incomplete	pSZL30.1	241247-248518	7.2	CP052818.1 (100/100)
SZL 31	S31-1	intact	chromosome	1683139-1740779	57.6	CP052817.1 (86/98)
	S31-2	intact	chromosome	2239713-2271755	32	CP052817.1 (100/100)
	S31-4	intact	pSZL31.1	31848-59439	27.5	CP052817.1 (100/100)
	S31-3	intact	chromosome	300-24896	24.5	CP052818.1 (100/100)
	S31-5	questionable	chromosome	1382924-1401553	18.6	CP052817.1 (100/100)
	S31-6	incomplete	chromosome	4518984-4539424	20.4	CP052817.1 (100/100)
	S31-7	incomplete	chromosome	2131116-2146365	15.2	CP052817.1 (100/100)
	S31-10	incomplete	chromosome	1242351-1256355	14.0	CP052817.1 (100/100)
	S31-13	incomplete	pSZL31.2	35434-47689	12.2	CP027138.1 (81/100)
	S31-8	incomplete	chromosome	3497522-3508122	10.6	CP052817.1 (100/100)
	S31-9	incomplete	chromosome	1753590-1761991	8.4	CP052817.1 (100/100)
	S31-11	incomplete	pSZL31.1	248954-256853	7.9	CP040066.1 (100/100)
	S31-12	incomplete	pSZL31.1	106784-114055	7.2	CP052818.1 (100/100)
SZL 38	S38-6	intact	pSZL38.1	517-79223	78.7	CP019188.1 (85/99)
	S38-1	intact	chromosome	1964077-2024744	60.6	CP050130.1 (100/100)
	S38-2	intact	chromosome	256927-314956	58	CP050726.1(100/100)
	S38-3	intact	chromosome	4624695-4670730	46	CP050726.1(100/100)
	S38-4	intact	chromosome	1247306-1286603	39.2	CP050726.1(100/100)
	S38-5	intact	chromosome	4556354-4593542	37.1	CP014659.2 (97/97)
	S38-7	questionable	chromosome	4514613-4558033	43.4	CP053294.1 (100/100)
	S38-8	questionable	chromosome	3959980-3998256	38.2	CP050726.1(100/100)
	S38-9	questionable	chromosome	418957-432910	13.9	CP050726.1(100/100)
	S38-10	incomplete	chromosome	3384024-3410360	26.3	CP043667.1 (99/99)
	S38-11	incomplete	chromosome	2881990-2902409	20.4	CP050726.1(100/100)
	S38-12	incomplete	chromosome	4910546-4919658	9.1	CP050726.1(100/100)
	S38-13	incomplete	chromosome	1356664-1363708	7.0	CP050726.1(100/100)
